# Integrative Functional Transcriptomic Analyses Implicate Shared Molecular Circuits in Sensorineural Hearing Loss

**DOI:** 10.3389/fncel.2022.857344

**Published:** 2022-03-14

**Authors:** Peng Chen, Jun-Jun Hao, Meng-Wen Li, Jing Bai, Yuan-Ting Guo, Zhen Liu, Peng Shi

**Affiliations:** ^1^State Key Laboratory of Genetic Resources and Evolution, Kunming Institute of Zoology, Chinese Academy of Sciences, Kunming, China; ^2^Kunming College of Life Science, University of Chinese Academy of Sciences, Kunming, China; ^3^School of Future Technology, University of Chinese Academy of Sciences, Beijing, China; ^4^Center for Excellence in Animal Evolution and Genetics, Chinese Academy of Sciences, Kunming, China

**Keywords:** sensorineural hearing loss, aging, noise exposure, ototoxic drugs, common molecular mechanisms, co-expression network

## Abstract

Sensorineural hearing loss (SNHL) is referred to as the most common type of hearing loss and typically occurs when the inner ear or the auditory nerve is damaged. Aging, noise exposure, and ototoxic drugs represent three main causes of SNHL, leading to substantial similarities in pathophysiological characteristics of cochlear degeneration. Although the common molecular mechanisms are widely assumed to underlie these similarities, its validity lacks systematic examination. To address this question, we generated three SNHL mouse models from aging, noise exposure, and cisplatin ototoxicity, respectively. Through constructing gene co-expression networks for the cochlear transcriptome data across different hearing-damaged stages, the three models are found to significantly correlate with each other in multiple gene co-expression modules that implicate distinct biological functions, including apoptosis, immune, inflammation, and ion transport. Bioinformatics analyses reveal several potential hub regulators, such as *IL1B* and *CCL2*, both of which are verified to contribute to apoptosis accompanied by the increase of (ROS) in *in vitro* model system. Our findings disentangle the shared molecular circuits across different types of SNHL, providing potential targets for the broad effective therapeutic agents in SNHL.

## Introduction

Sensorineural hearing loss (SNHL) refers to a type of hearing loss resulting from the structurally and functionally damaged inner ear and vestibulocochlear nerve, mainly including cochlear hair cells (HCs), stria vascularis (SV), and spiral ganglion neurons (SGNs) (Liberman and Kujawa, 2017). Compared to other kinds of hearing loss, SNHL is usually permanent and accounts for approximately 90% of reported hearing loss cases (Li et al., 2017). Sudden SNHL affects 5 to 27 per 100,000 people each year, with approximately 66,000 new annual cases in the United States (Alexander and Harris, 2013). Among various causes of SNHL, aging, noise exposure, and ototoxic drugs are widely believed to play predominant roles (Liberman and Kujawa, 2017; Wang and Puel, 2018). Worldwide, more than half of the population aged over 60 years suffer the age-related hearing loss (Gates and Mills, 2005) and ∼16% of the adults with hearing loss are attributed to occupational noise (Nelson et al., 2005). Ototoxicity is well-established toxicity associated with therapeutic agents for causing cochlear impairment and the prevalence of ototoxicity-induced hearing loss ranges from 4 to 90% in patients who have received the therapies with potential ototoxicity (Landier, 2016).

The three types of SNHL above display highly similar pathophysiological characteristics of the damaged or degenerated HCs, SV, and SGNs (Wang et al., 2007; Ding et al., 2012; Breglio et al., 2017; Keithley, 2020), suggesting shared biological mechanisms underlying the SNHL. Although most studies focus on one or two causative factors of SNHL, it is accessible to generalize the shared biological involvements by the clues from those independent studies (Yang et al., 2015; Cheng et al., 2019; Su et al., 2020; Maeda et al., 2021; Taukulis et al., 2021). For example, several biological processes have been suggested to influence apoptosis in cochleae affected by SNHL, such as mitochondrial dysfunction, oxidative stress, inflammation, and additional immunological reaction (Wang and Puel, 2018; Zhang et al., 2021), while autophagy may regulate the survival of HCs and SGNs in such inner ears affected by SNHL (Guo et al., 2021; He et al., 2021). Nevertheless, the validity of the shared biological processes, as well as the jointly involved genes, among different types of SNHL lacks systematic examination.

To address this question, we here created three SNHL mouse models based on aging, noise exposure, and cisplatin ototoxicity, and constructed their cochlear transcriptional co-expression networks during the generation of SNHL models. By identifying the modules with similar gene expression trajectories among different types of SNHL, we assessed the shared biological functions and signaling pathways among the genes involved in these modules, and experimentally confirmed the contributions of hub genes to mechanisms underlying phenotypic changes of SNHL.

## Results

### Generation of Three Sensorineural Hearing Loss Mouse Models Based on Aging, Noise Exposure, and Ototoxicity

**FIGURE 1:**
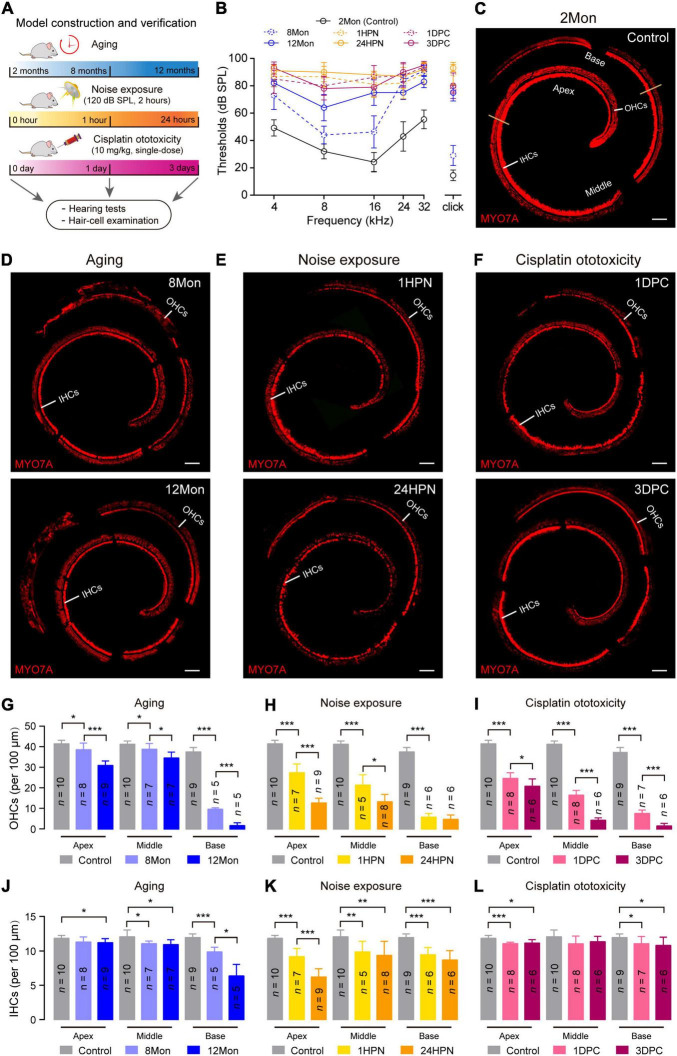
Representative phenotypic transformation in the three types of SNHL mouse models. **(A)** Flowchart of the overall approach to SNHL mouse models. 2 months, 8 months, and 12 months for the aging SNHL model are the actual ages of the mice, while one hour, 24 h, one day, and 3 days are the times after the respective intervention for the 2-month-old mice. **(B)** ABR audiogram in the three types of SNHL mouse models. ABR thresholds of wild type mice at 8 months (8Mon, *n* = 5, dashed-line in blue), 12 months (12Mon, *n* = 5, solid-line in blue), 1 hour post noise exposure (1HPN, *n* = 5, dashed-line in orange), 24 h post noise exposure (24HPN, *n* = 5, solid-line in orange), 1 day post cisplatin injection (1DPC, *n* = 4, dashed-line in purple), 3 days post cisplatin injection (3DPC, *n* = 5, solid-line in purple) show severe hearing loss, compared with wild type mice at 2 months (2Mon, *n* = 12, dashed-line in black). **(C–F)** Representative confocal microscopy images from whole-mount cochleae. The ears of aging and treatments with noise exposure and cisplatin injection exhibited substantial loss of hair cells compared with control ears. Scale bars, 100 μm. **(G–L)** Quantification of survival OHCs and IHCs in the three types of SNHL mouse models. The three types of SNHL mouse models are present in columns with different colors. *n* in the columns shows the numbers of cochleae used for the quantification. All data are shown as Mean ± SD. *P < 0.05, **P < 0.01, ***P < 0.001. *P* values are from two-tailed Student’s *t*-tests.

To systematically investigate the shared molecular circuits involved in SNHL, we generated three SNHL models in C57BL/6 mice strain from aging, noise exposure, and cisplatin ototoxicity, respectively (Figure 1A). The C57BL/6 mice present the hearing sensitivity reduction ∼6-month old and nearly complete deafness ∼18-month old, thus usually being regarded as an animal model of early-onset age-related hearing loss (Hequembourg and Liberman, 2001). Two-month-old C57BL/6 mice were used to generate other two SNHL models by being exposed to 120 decibel sound pressure level (dB SPL) noises for 2 h (Maeda et al., 2017), and by being intraperitoneally injected a single dose of cisplatin (10 mg/kg) and furosemide (200 mg/kg) (Wang et al., 2004; Li et al., 2011). Furosemide, as a potent loop diuretic, is often used to reduce the nephrotoxicity caused by cisplatin and enhance the entry of ototoxic drugs into the cochleae (Santoso et al., 2003; Ding et al., 2012).

To verify the successful establishment of the three SNHL models, we measured auditory brainstem responses (ABR) and examined the status of cochlear hair cells in the mice at 8- and 12-month age (8Mon and 12Mon), one- and 24-hour post noise exposure (1HPN and 24HPN), as well as one- and 3-day post cisplatin injection (1DPC and 3DPC), respectively (Figure 1A). Compared with the controls of 2-month-old (2Mon) C57BL/6 mice without any treatment, the ABR thresholds increased in 8Mon, 1HPN, and 1DPC mice, especially in 12Mon, 24HPN, and 3DPC mice at all examined frequencies (4, 8, 16, 24, and 32 kHz) and the broadband click stimulus (Figure 1B). *Myosin VIIa* (MYO7A), a cochlear hair-cell-specific marker, was used to stain the whole-mount cochleae of the three SNHL models (Figures 1C–F), indicating that the numbers of MYO7A-positive out hair cells (OHCs) significantly decreased in 8Mon, 1HPN, and 1DPC mice, and further in 12Mon, 24HPN, and 3DPC mice when compared with the controls (*P* < 0.05, two-tailed Student’ *t* tests; Figures 1G–I). It is notable that more damaged OHCs were observed at the basal region than at the apex when exposed to the excessive intense noises even with relatively low frequencies (120 dB SPL, 4-24 kHz), which may result from the more susceptibility of OHCs at the basal region as shown in the previous studies (Sanz et al., 2015; Miao et al., 2021). In addition, similar to OHCs, the inner hair cells (IHCs) were also generally lost during aging and after the treatments with noise exposure and cisplatin (Figures 1J–L). These similar phenotypic alterations of the reduced hearing sensitivity and the damaged cochlear hair cells suggest the successful establishment of the SNHL mouse models based on aging, noise exposure, and cisplatin ototoxicity.

### Transcriptome-Wide Gene Co-expression Networks Reflect Shared Molecular Circuits Among Different Sensorineural Hearing Loss Models

**FIGURE 2:**
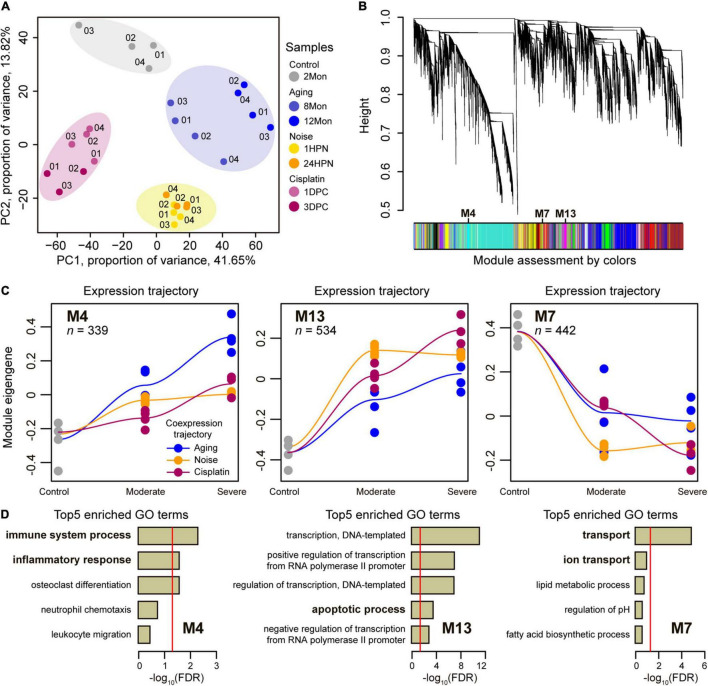
Co-expression network analysis. **(A)** Principal component analysis of cochlear RNA-seq samples in the triad of SNHL. The data revealed that the same SNHL model cochlear samples (represented by colors and labeled numerically) tended to cluster together. **(B)** Network analysis dendrogram showing modules based on the co-expression genes throughout the three types of SNHL mouse models. The colors below give information on module assessment. **(C)** Co-expression trajectory in the highlighted co-regulated modules, including two up-regulated modules (M4 and M13) and one down-regulated module (M7). Trajectories of the three types of SNHL mouse models were plotted by different colors. The fit line represents locally weighted scatterplot smoothing. *n* shows the numbers of the genes in each module. **(D)** Top 5 enriched GO terms of the highlighted co-regulated modules. The reports of –log_10_ (FDR) present relative enrichment in each module, with the red line at FDR = 0.05.

We reasoned that transcriptomic data of cochleae from different types of SNHL would inform our understanding of their shared molecular circuits, and thus examined gene expression dynamics during generating SNHL by sequencing cochleae transcriptomes across 2Mon, 8Mon, 12Mon, 1HPN, 24HPN, 1DPC, and 3DPC mice. To ensure the reliability of the data, at least three biological replicates were designed for each sample. We generated a total of 202.3 Gb clean data for cochlear transcriptomes across 27 samples from the above three types of SNHL models (Supplementary Table 1). The global relationships among these cochlear transcriptomes were explored through the principal components analysis (PCA). As expected, the samples from the same SNHL model tended to cluster together, suggesting strong commonalities and repeatability of transcriptomic data within each of the three SNHL mouse models (Figure 2A). We next performed signed weighted gene co-expression network analysis (WGCNA) (Langfelder and Horvath, 2008) for a total of 17,040 genes with available expression data across 27 samples and identified 22 co-expression modules labeled with colors and numbers (Figure 2B and Supplementary Table 2). The genes within each of these co-expression modules are expected to exhibit highly similar expression patterns during the generation of three types of SNHL models (Langfelder and Horvath, 2008). We investigated each module’s trajectory along with the stages of generating SNHL models by calculating the module eigengene (ME) (Supplementary Figure 2). The ME is the first principal component of a module and reflects the general expression pattern of the genes within the module (Langfelder and Horvath, 2008). We then calculated the Pearson correlation coefficients (*R*) of ME values for each module between the three types of SNHL above and identified three modules with larger averaged *R* values (> 0.92) than other modules (Figure 2C and Supplementary Table 3), including two up-regulated modules (M4 and M13) and one down-regulated module (M7). Consequently, the closely aligned gene co-expression patterns between the aging-, noise-, and cisplatin-induced SNHL were suggested in these three modules. Further, we, respectively, assessed biological functions among the genes within each of the three modules by enrichment for Gene Ontology (GO) annotation terms (Supplementary Tables 4–6). Top5 GO terms included immune system process and inflammatory response in M4, apoptotic process in M13, and transport and ion transport in M7 (Figure 2D), suggesting that the co-up-regulated genes involved in immune systems, inflammatory responses, and apoptotic process, as well as the co-down-regulated genes involved in transport and ion transport play critical roles on the occurrence of SNHL.

### Shared Signaling Pathways Associated With Sensorineural Hearing Loss

**FIGURE 3:**
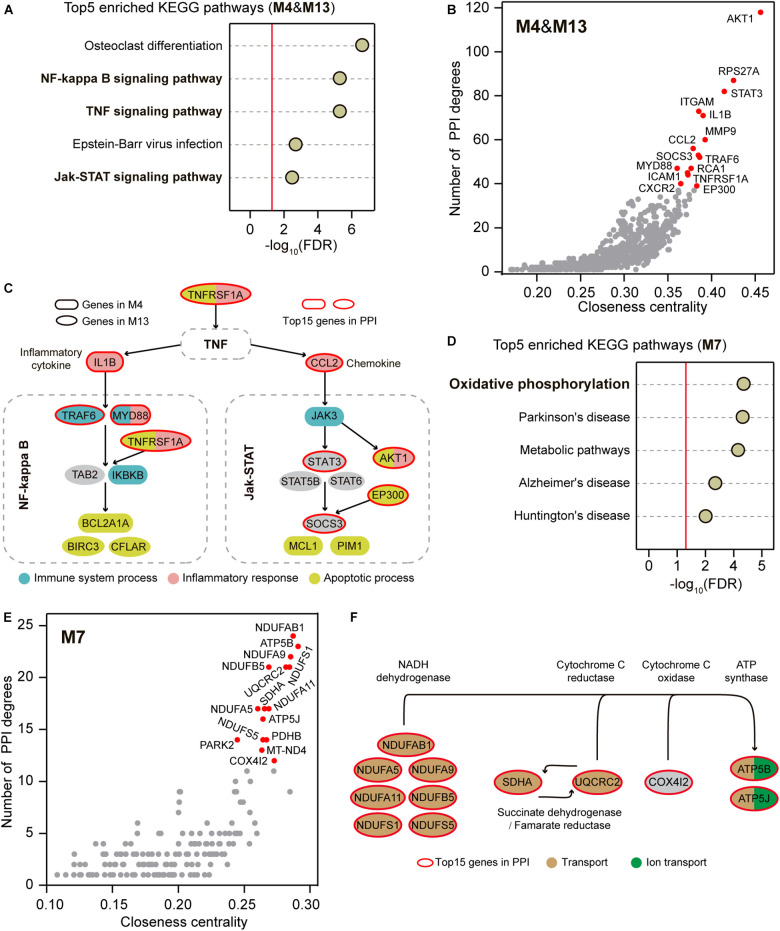
Signaling pathway enrichment and PPI analysis for the co-regulators. **(A)** Top 5 enriched KEGG pathways within M4 and M13. The reports of -log_10_ (FDR) present relative enrichment with the red line at FDR = 0.05. **(B)** PPI network analysis within M4 and M13. The top 15 highlighted genes are plotted in red. **(C)** Top 15 highlighted some representative genes of M4 and M13 that implicated in a cross-linked pathway composited with the enriched signaling pathways based on KEGG Mapper. The top 15 genes in PPI are marked in red circles and the genes implicated in immune system process, inflammatory response, and apoptotic process are plotted by different colors. **(D)** Top 5 enriched KEGG pathways within M7. The reports of –log_10_ (FDR) present relative enrichment with the red line at FDR = 0.05. **(E)** PPI network analysis within M7. The top 15 highlighted genes are plotted in red. **(F)** Top 15 highlighted genes of M7 that implicated in oxidative phosphorylation based on KEGG Mapper. The top 15 genes in PPI are marked in red circles and the genes implicated in transport and ion transport are plotted by different colors.

Although the immune system process, inflammatory response, and apoptotic process were enriched in two distinct up-regulated modules M4 and M13 (Figure 2D), these functionally different biological processes can be interactively aroused through some signaling pathways (Mak and Yeh, 2002; Simon, 2003; Fox et al., 2010). We thus hypothesized that shared signaling pathways or molecular regulatory relationships could link the genes within the two up-regulated modules. We first performed Kyoto Encyclopedia of Genes and Genomes (KEGG) enrichment analysis for the genes within M4 and M13. Of the top 5 enriched pathways, NF-kappa B signaling pathway, TNF signaling pathway, and Jak-STAT signaling pathway were found to implicate in immune, inflammatory, and apoptotic processes (Figure 3A and Supplementary Table 7). We next conducted the protein-protein interaction (PPI) analysis for the co-up-regulated genes within M4 and M13 to provide an independent line of interrogation for the centric profiles. We calculated the degree and the closeness centrality for each node, which are commonly used as centrality measures (Vital-Lopez et al., 2012), and examined their relationships for all nodes to identify hub genes in the network. The top 15 genes in the PPI network were highlighted, which directly interacted with 298 (41.5%) co-up-regulated genes and involved in 840 (22.9%) PPIs (Figure 3B). Notably, we found that 9 of the top 15 genes were involved in the NF-kappa B signaling pathway, TNF signaling pathway, and Jak-STAT signaling pathway, including *TNFRSF1A*, *IL1B*, *CCL2*, *MYD88*, *TRAF6*, *STAT3*, *EP300*, *SOCS3*, and *AKT1* (Figure 3C). Interestingly, nearly all of these genes (7/9) were associated with the biological functions of immune system process, inflammatory response, and apoptotic process that were determined by the GO enrichment analysis for M4 and M13 (Figure 3C).

Similarly, we performed the KEGG enrichment analysis for the genes within the down-regulated M7 and found that oxidative phosphorylation was the most notable KEGG term (Figure 3D and Supplementary Table 8). The top 15 genes in the PPI network constructed using the co-down-regulated genes in M7 were also highlighted (Figure 3E), which directly interacted with 61 (26.2%) co-down-regulated genes and involved 187 (43.2%) PPIs. Among the top 15, 12 genes were involved in mitochondria-related ATP production through oxidative phosphorylation, including *NDUFS1*, *NDUFS5*, *NDUFA5*, *NDUFA9*, *NDUFA11*, *NDUFAB1*, *NDUFB5*, *SDHA*, *UQCRC2*, *COX4I2*, *ATP5B*, and *ATP5J* (Figure 3F). Likewise, nearly all of these genes (11/12) were associated with the biological functions of transport and ion transport that were determined by the GO enrichment analysis for M7 (Figure 3F).

### Experimental Verification for Potential Contributions of *IL1B* and *CCL2* to Sensorineural Hearing Loss

Among the top 15 genes in the PPI network of the up-regulated modules M4 and M13, the pro-inflammatory cytokine *IL1B* and chemokine *CCL2* were found to serve as the connecting links between the TNF signaling pathway, NF-kappa B signaling pathway, and Jak-STAT signaling pathway (Figure 3C). Moreover, several previous studies show the increased expression of *IL1B* and *CCL2* after noise exposure, suggesting their crucial roles in generating noise-induced SNHL (Vethanayagam et al., 2016; Zhang et al., 2019; Wang et al., 2020). We next tested whether *IL1B* and *CCL2* also contribute to aging- and ototoxicity-induced SNHL using an *in vitro* model system.

The quantitative real-time polymerase chain reaction (qRT-PCR) was used to verify that both *IL1B* and *CCL2* were significantly up-regulated in the cochleae during creating different types of SNHL mouse models (Supplementary Figures 3A,B), as observed in the transcriptome-wide analyses. Next, we applied the house ear institute-organ of Corti 1 (HEI-OC1) cells as an *in vitro* model system for the aging- and ototoxicity-induced SNHL. The HEI-OC1 cells express several cochlear hair-cell-specific markers, such as *myosin VIIa*, *prestin*, *Atoh1*, *BDNF*, *calbindin*, and *calmodulin*, and thus are widely used for investigating the molecular mechanisms of the death or survival of cochlear hair cells (Kalinec et al., 2003, 2016).

**FIGURE 4:**
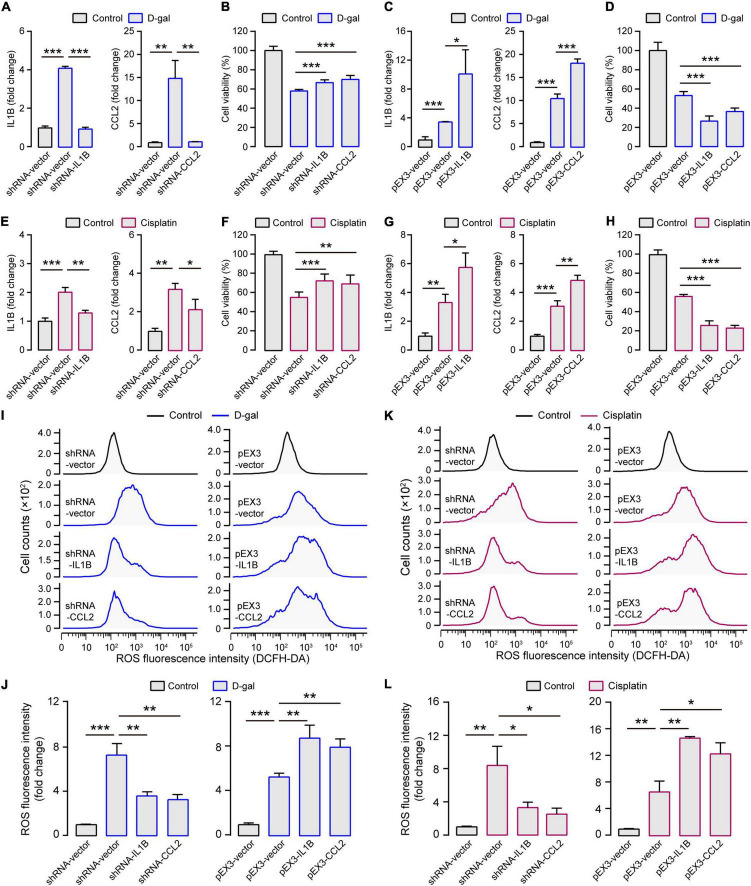
Experimental verification for contributions of *IL1B* and *CCL2* to SNHL. **(A)** In the aging *in vitro* model (treated with D-gal; shown in blue columns), the expression of *IL1B* and *CCL2* increased but decreased when shRNA-IL1B and shRNA-CCL2 were transfected. **(B)** The inhibited expression of *IL1B* and *CCL2* enhanced the cell viability in the aging *in vitro* model. **(C)** Both *IL1B* and *CCL2* overexpression could be elicited after pEX3-IL1B and pEX3-CCL2 transfections and **(D)** this further decreased the cell viability in the aging *in vitro* model. **(E)** In the ototoxicity *in vitro* model (treated with cisplatin; shown in purple columns), the expression of *IL1B* and *CCL2* increased but decreased when shRNA-IL1B and shRNA-CCL2 were transfected. **(F)** The inhibited expression of *IL1B* and *CCL2* enhanced the cell viability in the aging *in vitro* model. **(G)** Both *IL1B* and *CCL2* overexpression could be elicited after pEX3-IL1B and pEX3-CCL2 transfections and **(H)** this further decreased the cell viability in the ototoxicity *in vitro* model. **(I)** Representative cellular distribution with different ROS levels and **(J)** the quantification in *IL1B* and *CCL2* modulation groups in the aging *in vitro* model. **(K)** Representative cellular distribution with different ROS levels and **(L)** the quantification in *IL1B* and *CCL2* modulation groups in the ototoxicity *in vitro* model. In the two *in vitro* models, the levels of intracellular ROS decreased when *IL1B* or *CCL2* were inhibited and increased when *IL1B* or *CCL2* were overexpressed. The gene expression levels were measured by quantitative real-time PCR for each column (*n* = 3). The cell viability was measured by the cell proliferation assay kit for each column (*n* = 6). ROS evaluation was measured by the DCFH-DA kit using the Flow Cytometer for each column (*n* = 3). All the columns are present with Mean ± SD. *P < 0.05, **P < 0.01, ***P < 0.001. *P* values are from two-tailed Student’s *t*-tests.

Because D-galactose (D-gal) can increase oxidative stress, mitochondrial damage, and apoptosis, which accelerates tissue senescence, the treatment of HEI-OC1 cells with D-gal can largely mimic the alterations of cochlear hair cells during aging (Zhong et al., 2011; Du et al., 2012; He et al., 2020). The D-gal treatment decreased the viability of HEI-OC1 cells in concentration- and time-dependent manners (Supplementary Figures 4A,B), revealing that the half-lethal dose of D-gal was 75 mg/ml for 24 h, which was selected for generating the *in vitro* model of aged cochlear hair cells. As observed in the cochleae of aging-induced SNHL mice, both *IL1B* and *CCL2* were expressed significantly more in the HEI-OC1 cells treated with D-gal than without D-gal treatment (Figure 4A). When the expression of *IL1B* and *CCL2* was inhibited, the cell viability significantly increased (Figure 4B); when *IL1B* and *CCL2* were overexpressed, the cell viability significantly decreased (Figures 4C,D).

Similarly, we used the HEI-OC1 cells for testing the roles of *IL1B* and *CCL2* in generating ototoxicity-induced SNHL. The treatment with cisplatin also decreased the cell viability of HEI-OC1 cells in concentration- and time-dependent manners (Supplementary Figures 4C,D). The half-lethal dose of cisplatin (50 μM for 48 h) was used for generating the *in vitro* model of cisplatin-induced SNHL. Both *IL1B* and *CCL2* were expressed significantly more in the HEI-OC1 cells treated with cisplatin than without cisplatin treatment (Figure 4E). The inhibition of the expression of *IL1B* and *CCL2* increased the cell viability, but the overexpression of the *IL1B* and *CCL2* decreased the cell viability (Figures 4F–H). Notably, no cell viability changes were observed when *IL1B* and *CCL2* were inhibited and overexpressed in HEI-OC1 cells without any treatments (Supplementary Figures 5A,B). These highly consistent results from the *in vitro* systems of cochlear hair cells strongly support the crucial roles of *IL1B* and *CCL2* in generating the aging- and ototoxicity-induced SNHL.

The expression and activation of *IL1B* and *CCL2* have been reported to closely relate to the production of reactive oxygen species (ROS) (Li et al., 2014; Ansari et al., 2018). ROS can trigger and modulate inflammation and immune responses, and excessive cellular ROS production causes oxidative stress and mitochondrial abnormalities that contribute to the degenerative responsiveness to aging, intense noise, and ototoxic drugs in the auditory system (Someya and Prolla, 2010; Yu et al., 2014; Jing et al., 2015). To test whether the roles of *IL1B* and *CCL2* in generating SNHL are mediated by regulating ROS production, we examined ROS levels in HEI-OC1 cells with different expressions of *IL1B* and *CCL2*. The ROS levels were indeed significantly higher in HEI-OC1 cells treated with D-gal than without any treatments (Figures 4I,J). Moreover, the ROS levels significantly decreased when the expression of *IL1B* and *CCL2* was inhibited and increased when the expression of *IL1B* and *CCL2* was enhanced (Figures 4I,J). And the similar results were presented in the cisplatin treatment groups (Figures 4K,L). These results suggest a general causal link between ROS production and different types of SNHL, and that both *IL1B* and *CCL2* contribute to the production of ROS in different SNHL mouse models.

## Discussion

Prior work has documented several biological processes and genes underlying cell death in the cochleae of SNHL, including mitochondrial dysfunction, oxidative stress, inflammation, immune system (Wang and Puel, 2018; Zhang et al., 2021). Most of the studies, however, focused on one or two of the three most causative factors of SNHL, i.e., aging, noise exposure, and ototoxic drugs. In this study, we generated three SNHL mouse models derived from aging, noise exposure, and cisplatin ototoxicity, constructed gene co-expression modules of the cochlear transcriptomes during creating SNHL models and systematically validated the crucial roles of NF-kappa B signaling pathway, Jak-STAT signaling pathway, TNF signaling pathway, and Oxidative phosphorylation in generating SNHL. The *in vitro* experiments demonstrated that the overexpression of *IL1B* and *CCL2* in SNHL increased ROS production, likely leading to apoptosis in the cochleae of SNHL. Together, these findings clarify the shared molecular circuits and genes underlying different types of SNHL.

Besides *IL1B* and *CCL2*, several additional co-regulators were found to be involved in the SNHL-shared inflammatory and immune responses through TNF signaling pathway, NF-kappa B signaling pathway, and Jak-STAT signaling pathway, including *TNFRSF1A*, *TRAF6*, *MYD88*, *AKT1*, *STAT3*, *EP300*, and *SOCS3* (Figure 3C). Some of these genes have been identified to be up-regulated in the cochleae of a particular kind of SNHL. For example, the expression levels of *MYD88*, *AKT1*, and *SOCS3* increase after noise exposure (Gratton et al., 2011; Chen et al., 2015; Zhang et al., 2019), and exposure of cochlear explants to the ototoxic drug gentamicin leads to the increased *TNFRSF1A* expression (Bas et al., 2012). Interestingly, some of these regulators are suggested to be recognized as potential targets for ameliorating SNHL. The treatment with IL-1 blockade improves the hearing of 91% of Muckle-Wells-syndrome patients (Kuemmerle-Deschner et al., 2015). Avenanthramide-C reduces *IL1B* and *TNF-*α expression and provides significant protection against noise- and drug-induced SNHL (Umugire et al., 2019). Capsaicin protects against cisplatin-induced SNHL by changing the STAT3/STAT1 expression ratio (Bhatta et al., 2019). Our findings extend the current understanding of the shared biological processes and hub genes in SNHL and provide the elevated probability of discovering the most effective therapeutic and preventive targets across different types of SNHL.

In addition to the shared molecular circuits and regulators, we also identified some prime modules with specific co-expression patterns for each of the three SNHL mouse models. For instance, M6 reflected a down-regulated gene co-expression pattern for the aging-induced SNHL model compared to the noise- and ototoxicity-induced SNHL models. Among the top 5 enriched GO terms for the genes within M6, there were four involved in the regulation of gene transcription (Supplementary Figure 6A and Supplementary Table 9). M11 and M14, respectively, displayed down- and up-regulated co-expression patterns for the noise-induced SNHL model. Despite no significantly enriched GO terms for the genes within M11, the enriched GO terms for M14 included angiogenesis and steroid metabolic (Supplementary Figure 6B and Supplementary Table 10). M16 and M21 both presented down-regulated co-expression patterns for the cisplatin-induced SNHL model; the notable GO terms included cell adhesion, immune system process, cell cycle, and cell division (Supplementary Figure 6C and Supplementary Table 11). These functional involvements have been shown implications for the normal functioning of cochleae more or less (Malgrange et al., 2015; Wang et al., 2019); however, more research is needed before their causal roles in generating SNHL from specific factors is known for certain.

Although we acknowledge that the stages in the three types of SNHL selected for comparison may be artificial, the approaches for shared molecular circuits are reinforced. Our analyses systematically provide evidence for existing the shared molecular circuits in different types of SNHL and highlight the importance of future investigation for the shared molecular circuits in SNHL. Regardless, our analyses suggest some common potential targets of the most effective therapeutic agents for preventing or ameliorating aging-, noise-, and ototoxicity-induced SNHL.

## Materials and Methods

### Animals

C57BL/6 mice were obtained from the Kunming Institute of Zoology Southwest SPF Animal Center. All animal experiments were performed under Animal Use Protocols approved by the Kunming Institute of Zoology Animal Care and Ethics Committee, CAS.

### Acoustic Overexposure

Two-month-old mice were continuously exposed to broadband noises (4-24 kHz) at 120 -121 decibel Sound Pressure Level (dB SPL) for 2 h in a small cylindrical cage (706.5 cm^2^ × 15 cm.). The noise was delivered by a loudspeaker (HG10044XT; Weijie-Electric, Guangzhou, CN) at a distance of 20 cm above the bottom of the cage. Calibration of noise to target SPL was performed immediately before each noise exposure session to ensure that the SPL varied by < 1 dB across the cage.

### Cisplatin Treatment

Two-month-old mice were treated with co-administration of furosemide and cisplatin. All the treatments were performed by intraperitoneal injection. The 200 mg/kg of furosemide (Hongbao, CN) treatment was administered one hour before being intraperitoneally injected with 10 mg/kg of cisplatin (P4394, Sigma- Aldrich). Mice were daily performed by intraperitoneal injection with 1 ml of normal saline for the following 3 days.

### Auditory Brainstem Response Measurement

The anesthetized animals with an intraperitoneal injection of sodium pentobarbital (90 mg/kg) were placed on an anti-vibration table in a soundproof room. The recording electrode (a subdermal needle) was inserted at the skull vertex and the reference electrode was placed on a 1-2 mm incision ventroposterior to the external pinna. During ABR recordings, the animal’s body temperature was maintained at 37.5°C by a heating pad. Click or tone burst sounds (4, 8, 16, 24, and 32 kHz) with 5 ms duration were delivered from 10 to 90 dB SPL with 5 dB interval at a rate of 10 per second by a calibrated MF1 speaker (TuckerDavis Technologies, Alachua, FL) which was placed ∼2 cm in front of the animal. After being amplified, filtered (100-1000 Hz), and averaged (256 times) by an RZ6 Processor (TuckerDavis Technologies Alachua, FL), the ABR signals were recorded using BioSigRZ software (TuckerDavis Technologies, Alachua, FL). The minimum sound intensity that could elicit a detectable response was defined as the ABR threshold. If no detectable ABR waveforms, the ABR thresholds were arbitrarily defined as 95 dB SPL for statistical analysis.

### Cochlear Pathology

After mice were killed with CO_2_ inhalation, the cochleae were collected and fixed in 4% paraformaldehyde (PFA) at 4°C overnight and decalcified in 10% ethylene diamine tetraacetic acid (EDTA) solution at room temperature for several days. Then, the cochleae were divided into pieces for whole-mount immunofluorescence. After being infiltrated with 0.3% Triton X-100 (Sigma-Aldrich) for 20 min and blocked with 10% goat serum for one hour, the tissues were applied with rabbit anti-MYO7A (1:500; Proteus BioSciences) at 4°C overnight. After three rinses with PBS, the tissues were incubated in goat anti-rabbit Alexa Fluor 568 (1:2000; Invitrogen) for one hour at room temperature. The samples were mounted in an antifading mountant medium (Cat.#. S2110, Solarbio) after three rinses with PBS. With maximum intensity projections of z-stacks, the confocal images of cochlear hair cells were taken by a microscope (Nikon A1) using a 10 × lens. The numbers of MYO7A-positive cells along 500 μm in each section were counted for statistical analysis. The composite images showing the whole cochlea were constructed by using Adobe Photoshop CC 2019 and Adobe Illustrator CC 2019.

### Collection of Cochlear Total RNA

The cochleae were harvested from controls (2-month-old mice), aging-induced SNHL (8-month-old and 12-month-old mice), noise-induced SNHL (2-month-old mice after being exposed to noise for one hour and for 24 h), and cisplatin-induced SNHL (2-month-old mice after being injected with cisplatin for one day and for three days). An individual’s bilateral cochleae were collected as a biological replicate. To ensure the reliability and repeatability of our data, at least three replicates were required. After removing the vestibule organ in a Petri dish filled with ice-cold sample protector for RNA/DNA (Cat.#. 9750, TaKaRa), we extracted the total RNA from the remaining cochleae using QIAzol Lysis Reagent (Qiagen Science). The quality and integrity of the purified total RNA with the RNeasy Plus Universal Mini Kit (Cat.#. 73404, Qiagen Science) were examined using Agilent 2100 Bioanalyzer.

### Transcriptome Sequencing and Analysis

Total RNA was qualified and quantified using a NanoDrop and Agilent 2100 bioanalyzer (Thermo Fisher Scientific, MA, United States). Approximately 1 μg of total RNA was used to construct cDNA libraries according to the manufacturer’s recommendations. All the libraries were sequenced on the MGISEQ2000 platform (BGI-Shenzhen, China) in a paired-end form with 150 bp. The low-quality reads were filtered out using the program of the fastq-quality-filter (from Fastx-Toolkit 0.0.13) with the parameters of -Q 33 -q 20 -p 80. A total of ∼202.3 Gb clean data was obtained and averaged 7.49 Gb high-quality clean reads for 27 samples (Supplementary Table 1). The clean reads were mapped onto the mouse genome (version GRCm38) using Tophat 2.1.0 (Kim et al., 2013) with the parameter of –read-mismatches 2. And then, the expected fragments per kilobase of transcript per million fragments (FPKM) of each gene was calculated using cufflinks 2.02 (Trapnell et al., 2010) with the parameter –max-multiread-fraction 0.75 as its expression level. The raw sequence data reported have been deposited both in the Genome Sequence Archive in National Genomics Data Center, China (CRA005119)^1^ and in the Gene Expression Omnibus in National Center for Biotechnology Information (GSE196870)^2^.

### Weighted Gene Co-expression Network Analysis

The *R* package of signed weighted gene co-expression network analysis (WGCNA) was used to construct gene co-expression networks (Langfelder and Horvath, 2008). If a gene has > 0.5 FPKM values in less than half of 27 samples, it will be discarded. Finally, a total of 17,040 genes were retained and their FPKM values were logarithmically transformed [log_2_(FPKM + 1)] to generate an integrated expression matrix for constructing co-expression networks with WGCNA. Based on the best soft threshold (power = 15; Supplementary Figure 1), a total of 22 co-expression modules across all samples were identified (labeled by color and numerically, Supplementary Table 2).

### Module Assignment for Shared Molecular Regulation

We investigated each module’s trajectory following the stages of SNHL models by calculating the module eigengene (ME), which is the first principal component of a module and reflects the general expression pattern of the genes within the module (Supplementary Figure 2). To determine the shared molecular regulation across the three types of SNHL, we calculated the Pearson correlation coefficients (*R*) of ME values for each module between the three types of SNHL induced by aging, noise exposure, and cisplatin injection, respectively (Supplementary Table 3). The modules with *R* > 0.85 in any two of the three types of SNHL were considered as closely aligned gene co-expression patterns for shared molecular regulation.

### Functional and Pathway Enrichment Analyses and Constructing Protein-Protein Interaction (PPI) Networks

The mouse Gene Ontology (GO) annotations and Kyoto Encyclopedia of Genes and Genomes (KEGG) pathways downloaded from DAVID Bioinformatics Resources 6.8 (david.ncifcrf.gov) were used to assign functional categories to one-to-one orthologous genes. Using all mouse genes as the background, we applied an in-house Fisher’s exact test program to perform the statistical analysis. The obtained *P* values were corrected by the false discovery rate (FDR). The maps of the related KEGG pathways were obtained from its official website^3^. We constructed the PPI network using STRING version 11.0^4^. The number of degrees and the closeness centrality value for each gene involved in the networks were calculated by the software Cytoscape 3.8.0 (Shannon et al., 2003).

### Cell Culture

The HEI-OC1 cells (Sigma-Aldrich, St. Louis, MO) were cultured in Dulbecco’s Modified Eagle’s Medium (Gibco, Thermo Fisher) containing 10% fetal bovine serum (Gibco, Thermo Fisher) without antibiotics at 33°C and 5% CO_2_. Cells were seeded in a 10 cm plate at a density of 5.0 × 10^5^ cells or a 96-well plate at a density of 1.0 × 10^4^ cells per well and incubated overnight for the next experiments.

### Quantitative Real-Time PCR

For Quantitative Real-Time PCR (qRT-PCR), the cochleae of two mice were pooled together to extract total RNA for the SNHL mouse models; the HEI-OC1 cells in a 10 cm plate were collected to extract total RNA for the *in vitro* experiments. The extracted total RNA was reverse transcribed into cDNA using a PrimeScript RT reagent kit with gDNA Eraser (Cat.#. RR047A, Takara, JP). The qRT-PCR assays were performed with GoTaq qPCR Master Mix (Cat.#. A6001, Promega, United States) using Quant-studio 12K Flex (AB Life Technologies), following the conditions: 95°C, 10 min; 95°C, 15 s; 60°C, 1 min; for 40 cycles; 95°C, 15 s; 60°C, 1 min; 95°C 15 s. qRT-PCR data were calculated with the 2^–ΔΔCt^ method and *GAPDH* was used as an endogenous reference control. Primer sequences used in this study were as follows: *IL1B*: Forward 5′- GAGTGTGGATCCCAAGCA AT-3′, Reverse 5′- ACGGAT TCCATGGTGAAGTC-3′; *CCL2*: Forward 5′- TTA AAAACCTGGATCGGAACCAA-3′, Reverse 5′- GCATTAGCT TCAGATTTACGGGT-3′; *GAPDH*: Forward 5′- ACCACCAT GGAGAAGGCC-3′, Reverse 5′- ATTGCTGACAATCTTGAGT GAGT-3′.

### Short Hairpin RNA and Plasmid cDNA Transfection

The HEI-OC1 cells in a 10 cm plate were transfected with 10 μg shRNA plasmid or cDNA plasmid in 45 μl of the Lipofectamine 3000, 30 μl P3000, 750 μl Opti-MEM, and 10 ml complete growth medium. The HEI-OC1 cells in a 96-well plate were transfected with 0.2 μg shRNA plasmid or cDNA plasmid in 0.3 μl of the Lipofectamine 3000, 0.2 μl P3000, 10 μl Opti-MEM, and 100 μl complete growth medium. The respective empty plasmid vectors were transfected as controls. After 48 h of transfection, the compound medium was removed and replaced by a complete growth medium with D-galactose or cisplatin. The shRNA-IL1B and shRNA-CCL2 plasmids (GenePharma, CN) were designed to knock down the expression of target genes. The cDNA plasmids of pEX3-IL1B and pEX3-CCL2 (GenePharma, CN) were designed to enhance the gene expression. The target sequence of shRNA is as follows: *IL1B*: 5′-GGACCCATATGAGCTGAAAGC-3′; *CCL2*: 5′-CACCAGCAAGATGATCCCAAT-3′.

### Assessment of Cell Viability

The HEI-OC1 cells in a 96-well plate were used for assessing cell viability with Celltiter 96 AQueous One Solution Cell Proliferation Assay (G3581, Promega). After transfection, the cells were treated with cisplatin (P4394, Sigma-Aldrich) or D-galactose (Coolaber, CN) at the indicated concentrations and the designed concentration for indicated periods. After the treatment with drugs, 10 μl compound was added for 2 h. Then, the optical density (OD) values were measured at 450 nm by a Hybrid reader (Synergy H1). The positive control underwent the same procedure without cell-seeding. The averaged OD in negative control cells was taken as 100% of viability. The relative viability was calculated as (OD experiment - OD positive)/(OD negative – OD positive) × 100. The negative control was the HEI-OC1 cells without any treatment.

### Measurement of Reactive Oxygen Species Levels

The cellular Reactive Oxygen Species (ROS) levels were measured by 2,7-Dichlorodihydrofluorescein diacetate (DCFH-DA; GK3611, Genview, CN) staining according to the manufacturer’s instructions. The HEI-OC1 cells were incubated with 10 μM DCFH-DA in DMEM for one hour and then washed twice with DMEM. The cells were collected to measure the ROS fluorescent signal intensity by flow cytometry (LSR Fortessa, Becton Dickinson, United States). Flow cytometry analyses were performed with extinction at 502 nm and emission at 530 nm (10,000 cells per sample) and then evaluated with the software FlowJo V10.

## Data Availability Statement

The original contributions presented in the study are publicly available. This data can be found here: https://ngdc.cncb.ac.cn/gsa, CRA005119. www.ncbi.nlm.nih.gov/geo/, GSE196870.

## Ethics Statement

The animal study was reviewed and approved by Kunming Institute of Zoology Animal Care and Ethics Committee, CAS.

## Author Contributions

PC, ZL, and PS designed the project. PC performed ABR recordings and cochleae staining. PC and JB prepared mice and cochlear samples. PC and M-WL performed the qRT-PCR and cell culture experiments. J-JH analyzed the RNA-seq data. Y-TG performed statistical tests. ZL, PC, and PS wrote the manuscript with input from all authors.

## Conflict of Interest

The authors declare that the research was conducted in the absence of any commercial or financial relationships that could be construed as a potential conflict of interest.

## Publisher’s Note

All claims expressed in this article are solely those of the authors and do not necessarily represent those of their affiliated organizations, or those of the publisher, the editors and the reviewers. Any product that may be evaluated in this article, or claim that may be made by its manufacturer, is not guaranteed or endorsed by the publisher.

## Footnotes

1
https://ngdc.cncb.ac.cn/gsa

2
www.ncbi.nlm.nih.gov/geo/

3
www.kegg.jp/kegg/tool/map_pathway2.html

4
string-db.org/cgi/input
