# Supplementary material for: Integrative Functional Transcriptomic Analyses Implicate Shared Molecular Circuits in Sensorineural Hearing Loss
Source: Front Cell Neurosci. 2022 Mar 14;16:857344. doi: 10.3389/fncel.2022.857344 (PMC8964368; doi:10.3389/fncel.2022.857344)
Supplement: Supplementary file 2 [file Data_Sheet_2.pdf]

Supplementary Figures

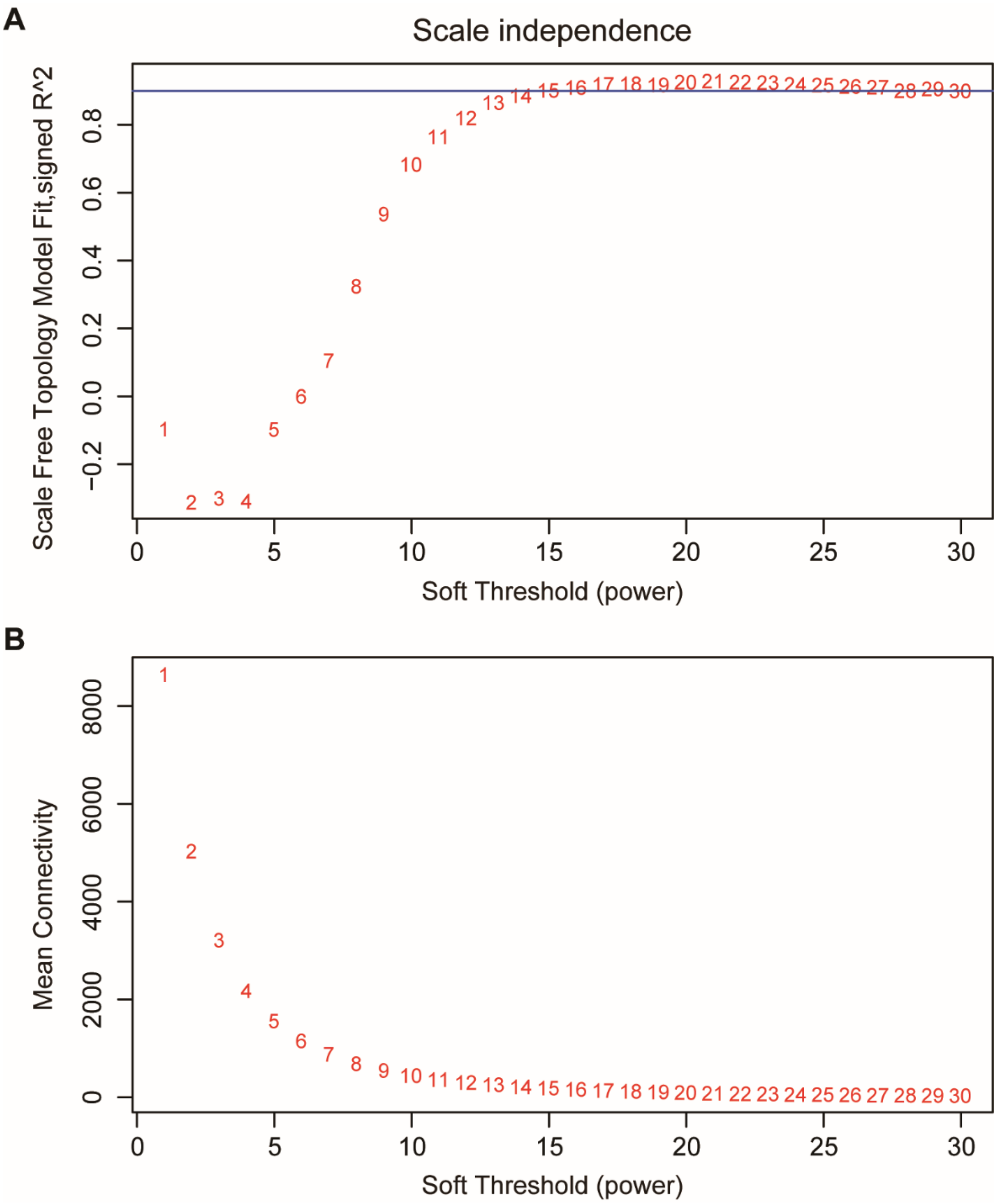

Supplemental Figure 1. Scale-free topology fit index and mean connectivity as a function of the soft-thresholding power. Scale independence **(A)** and mean connectivity **(B)** indicate 15 as the soft threshold. The blue line shows $R^2 = 0.9$.

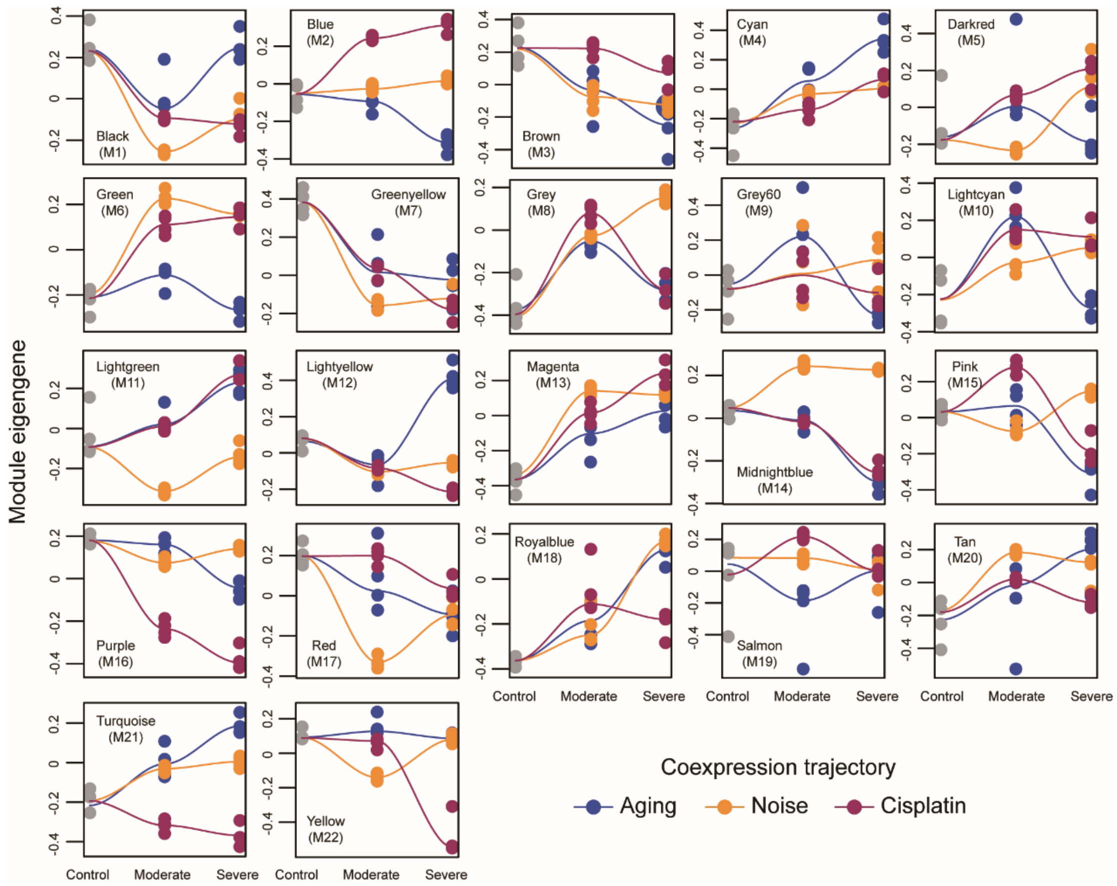

Supplemental Figure 2. The coexpression trajectories of the 22 modules. The WGCNA yielded 22 modules represented by colors and we relabeled them numerically. The coexpression trajectories during aging-, noise-, and cisplatin-induced hearing loss were plotted by different colors. The fit line represents locally weighted scatterplot smoothing.

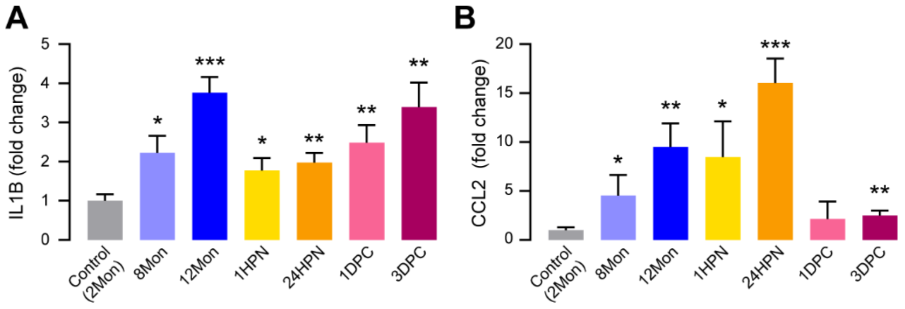

Supplemental Figure 3. qRT-PCR verification for *IL1B* **(A)** and *CCL2* **(B)** mRNA levels in the cochleae from the three types of SNHL mouse models. The three types of SNHL mouse models are present in columns with different colors. Mon: months; HPN: hour post noise exposure; DPC: day post cisplatin injection. All the columns are present with Mean ± SD, $n = 3$ for each column. * $P < 0.05$, ** $P < 0.01$, *** $P < 0.001$. P-value is calculated by the two-tailed Student's t-test.

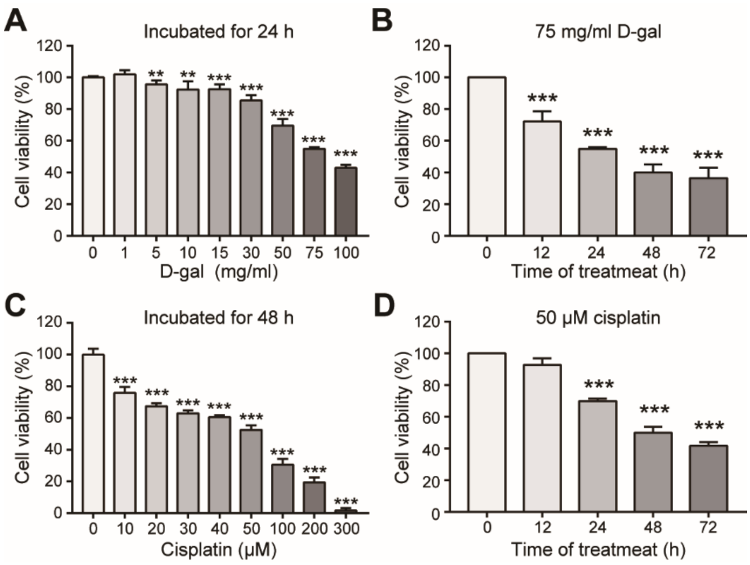

Supplemental Figure 4. Establishment for *in vitro* aging and ototoxicity models in HEI-OC1 cells. **(A)** Cell viability after the treatments with the designated concentration of D-gal for 24 h. **(B)** Cell viability after the treatments with 75 mg/ml D-gal for the designated periods. **(C)** Cell viability after the treatments with the designated concentration of cisplatin for 48 h. **(D)** Cell viability after the treatments with 50 μM cisplatin for the designated periods. Cell viability was measured by the cell proliferation assay kit. All the columns are present with Mean ± SD, $n = 6$ for each column. ** $P < 0.01$, *** $P < 0.001$. P-value is calculated by the two-tailed Student's t-test.

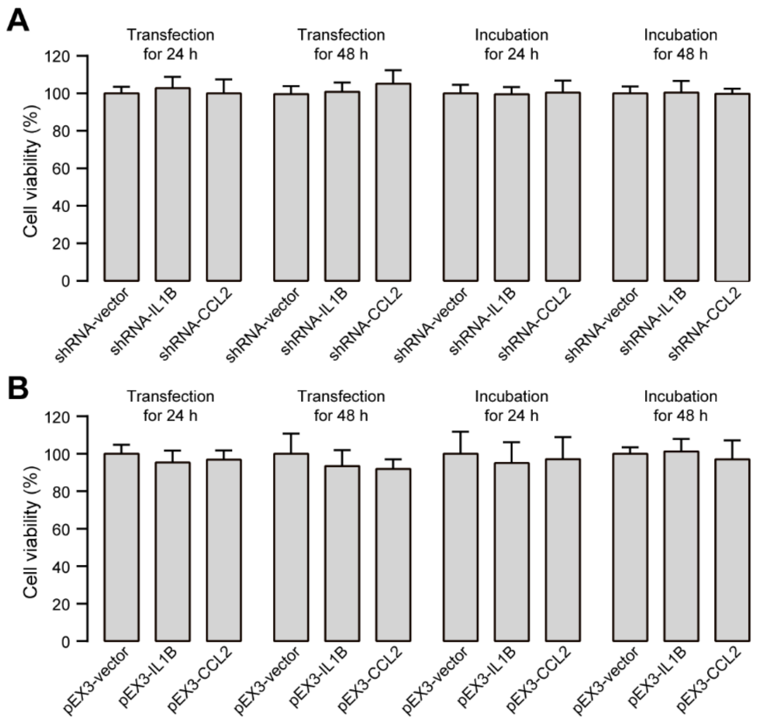

Supplemental Figure 5. Cell viability in HEI-OC1 cells after modulation of *IL1B* and *CCL2*. **(A)** Cell viability after the treatments with shRNA-IL1B and shRNA-CCL2 transfection (24 h and 48 h) and the following incubation (24 h and 48 h). **(B)** Cell viability after the treatments with pEX3-IL1B and pEX3-CCL2 transfection (24 h and 48 h) and the following incubation (24 h and 48 h). Cell viability was measured by the cell proliferation assay kit. All the columns are present with Mean ± SD, n = 6 for each column. P-value is calculated by the two-tailed Student's t-test.

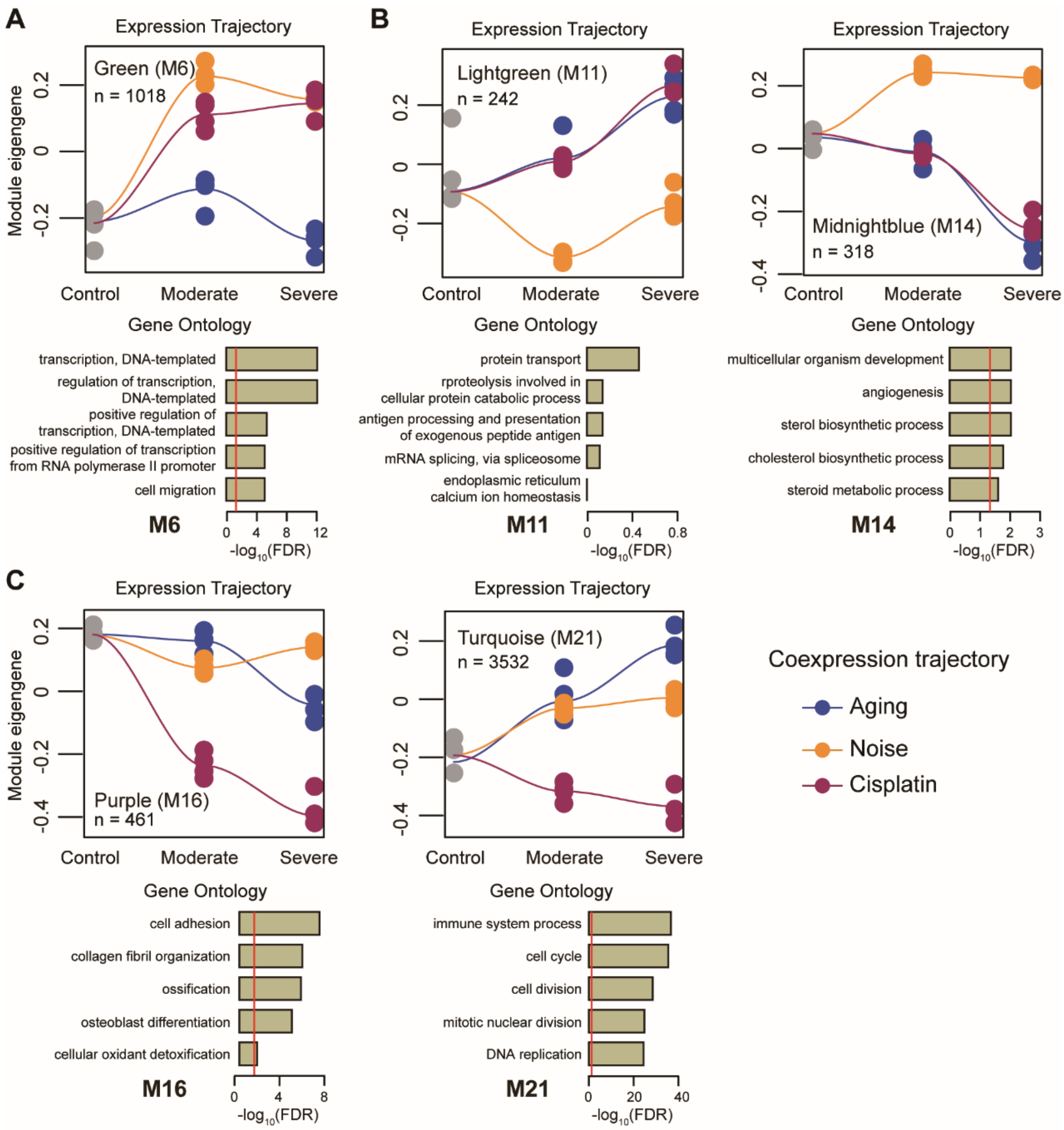

Supplemental Figure 6. Representative modules and their Top5 GO enrichments refer to aging-, noise-, and ototoxicity-induced SNHL independence. **(A)** M6 refers to aging-induced SNHL independence. **(B)** M11 and M14 refer to noise-induced SNHL independence. **(C)** M16 and M21 refer to cisplatin-induced SNHL independence. Coexpression trajectories of aging, noise, and cisplatin models were plotted by different colors. The fit line represents locally weighted scatterplot smoothing. n shows the numbers of the genes in each module. The reports of -log10 (FDR) resent relative enrichment in each module, with the red line at FDR = 0.05.
